# Diversity, Cyanotoxin Production, and Bioactivities of Cyanobacteria Isolated from Freshwaters of Greece

**DOI:** 10.3390/toxins11080436

**Published:** 2019-07-25

**Authors:** Spyros Gkelis, Manthos Panou, Despoina Konstantinou, Panagiotis Apostolidis, Antonia Kasampali, Sofia Papadimitriou, Dominiki Kati, Giorgia Maria Di Lorenzo, Stamatia Ioakeim, Sevasti-Kiriaki Zervou, Christophoros Christophoridis, Theodoros M. Triantis, Triantafyllos Kaloudis, Anastasia Hiskia, Minas Arsenakis

**Affiliations:** 1 Department of Botany, School of Biology, Aristotle University of Thessaloniki, GR-541 24 Thessaloniki, Greece; 2 Department of Genetics, Development and Molecular Biology, School of Biology, Aristotle University of Thessaloniki, GR-541 24 Thessaloniki, Greece; 3 Laboratory of Photo-Catalytic Processes and Environmental Chemistry, Institute of Nanoscience & Nanotechnology, National Center for Scientific Research “Demokritos”, Patriarchou Grigoriou & Neapoleos, Agia Paraskevi, 15341 Athens, Greece

**Keywords:** polyphasic taxonomy, antibacterial, cytotoxicity, microcystins, *Jaaginema*, *Synechococcus*

## Abstract

Cyanobacteria are a diverse group of photosynthetic Gram-negative bacteria that produce an array of secondary compounds with selective bioactivity against a broad spectrum of organisms and cell lines. In this study, 29 strains isolated from freshwaters in Greece were classified using a polyphasic approach and assigned to Chroococcales, Synechococcales, and Nostocales, representing 11 genera and 17 taxa. There were good agreements between 16S ribosomal RNA (rRNA)–*cpcBA*–internal genetic spacer (IGS) characterization and morphological features, except for the *Jaaginema*–*Limnothrix* group which appears intermixed and needs further elucidation. Methanol extracts of the strains were analyzed for cyanotoxin production and tested against pathogenic bacteria species and several cancer cell lines. We report for the first time a *Nostoc oryzae* strain isolated from rice fields capable of producing microcystins (MCs) and a *Chlorogloeopsis fritschii* strain isolated from the plankton of a lake, suggesting that this species may also occur in freshwater temperate habitats. Strains with very high or identical 16S rRNA gene sequences displayed different antibacterial and cytotoxic activities. Extracts from *Synechococcus* cf. *nidulans* showed the most potent antibacterial activity against *Staphylococcus aureus*, whereas *Jaaginema* sp. strains exhibited potent cytotoxic activities against human colorectal adenocarcinoma and hepatocellular carcinoma cells. *Jaaginema* Thessaloniki Aristotle University Microalgae and Cyanobacteria (TAU-MAC) 0110 and 0210 strains caused pronounced changes in the actin network and triggered the formation of numerous lipid droplets in hepatocellular carcinoma and green monkey kidney cells, suggesting oxidative stress and/or mitochondrial damage leading to apoptosis.

## 1. Introduction

Cyanobacteria are an ancient lineage of photosynthetic prokaryotes and important components of microbial communities of a wide range of habitats, including extreme environments. They are known for the production of toxins in freshwater ecosystems worldwide, which pose a threat to higher animals’ health [1]. These cyanotoxins can be classified into five broad groups according to their toxicity: hepatotoxins, neurotoxins, cytotoxins, dermatotoxins, and irritant toxins (lipopolysaccharides) [1,2], with the most frequent being the hepatotoxins, microcystins (MCs) [3]. While the majority of early research on cyanobacteria bioactive metabolites focused on toxin production, it is currently well known that marine, terrestrial, and freshwater cyanobacteria produce a wide variety of secondary metabolites with interesting biological activities [4,5,6].

The diversity in secondary metabolites arises from the cyanobacterial capacity to integrate both non-ribosomal peptide synthetases with polyketide synthases in their biosynthetic pathways [7]. Every single cyanobacterial strain is capable of producing a spectrum of secondary metabolites with unique chemical arrangements and interesting bioactivities [5]. A recent review revealed that the known chemical diversity of cyanobacterial natural products exceeds 1100 secondary metabolites with highly complex chemical structures; more than two-thirds of these are reported from the marine *Lyngbya*, the freshwater *Microcystis* and *Nostoc*, and the terrestrial *Hapalosiphon* [7]. These structural diverse substances have promising therapeutic potential, with anticancer, multidrug-reversing, antifungal, antibacterial, anti-inflammatory, antiviral, and potent enzyme-inhibiting bioactivities [8,9]. For instance, to date, more than 40 anticancer biomolecules were characterized from cyanobacteria [10], such as cryptophycins and curacins.

Concerning the unique biological activities of their natural products, cyanobacteria gathered considerable attention as potential source for drug development [11,12]. Several cyanobacterial natural products are in clinical or preclinical trials or undergoing further investigation (i.e., References [13,14,15]). In addition, ongoing research is screening more and more strains, showing that unknown secondary metabolites with valuable features for drug design still exist (i.e., References [16,17,18]). The majority of these recent studies testing the activity of cyanobacterial compounds and extracts is focused on marine cyanobacteria strains (i.e., References [18,19,20]), while less attention is given to freshwater strains.

Greek freshwater ecosystems appear to host a high diversity of cyanobacteria according to the species list that was recently published [21]. In a previous study, 29 planktic and benthic cyanobacteria strains were isolated from several Greek freshwater bodies [22]. Herein, we (a) characterize 29 cyanobacteria strains isolated from freshwaters, based on a polyphasic approach, (b) assess their potential toxicity with chemical methods, and (c) investigate the biological activity of their extracts using antibacterial and cytotoxicity assays.

## 2. Results

### 2.1. Polyphasic Taxonomy

**Figure 1:**
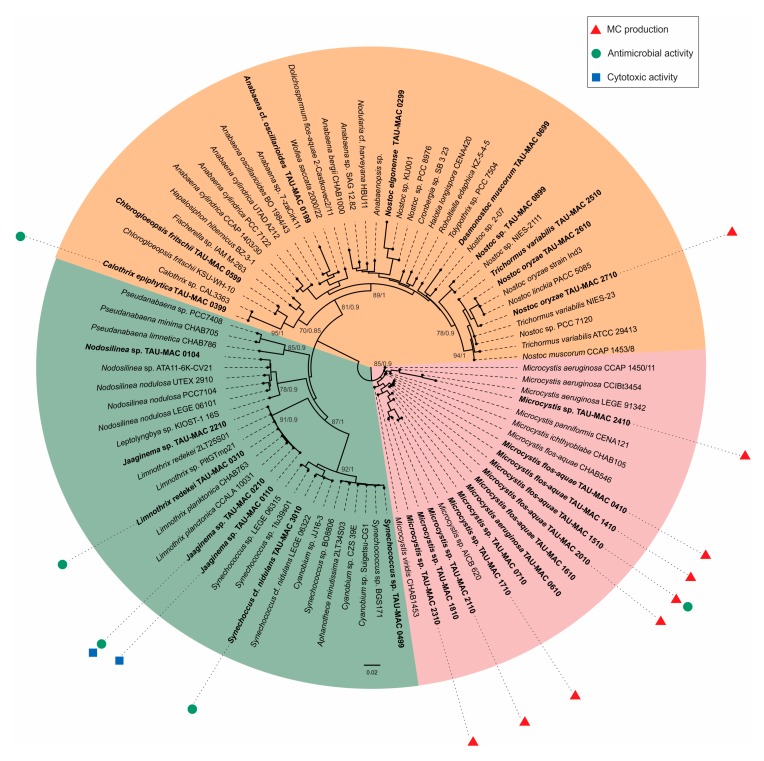
Phylogenetic tree based on 16S–23S ribosomal RNA (rRNA) and *cpcBA*–internal genetic spacer (IGS) sequences of Thessaloniki Aristotle University Microalgae and Cyanobacteria (TAU-MAC) strains and reconstructed using the maximum-likelihood (ML) method and Bayesian inference (BI) analysis. ML topology is demonstrated. Numbers above branches indicate the bootstrap value (as percentages of 1000 replications) for the ML method and the posterior probabilities for the BI method. Strains of the present study are indicated in bold. Red triangles represent microcystins, green dots represent antibacterial activity, and blue squares represent cytotoxic activity. GenBank accession numbers are indicated in Table S2 (Supplementary Materials). The bar represents 0.020 nucleotide substitutions per site.

**Figure 2:**
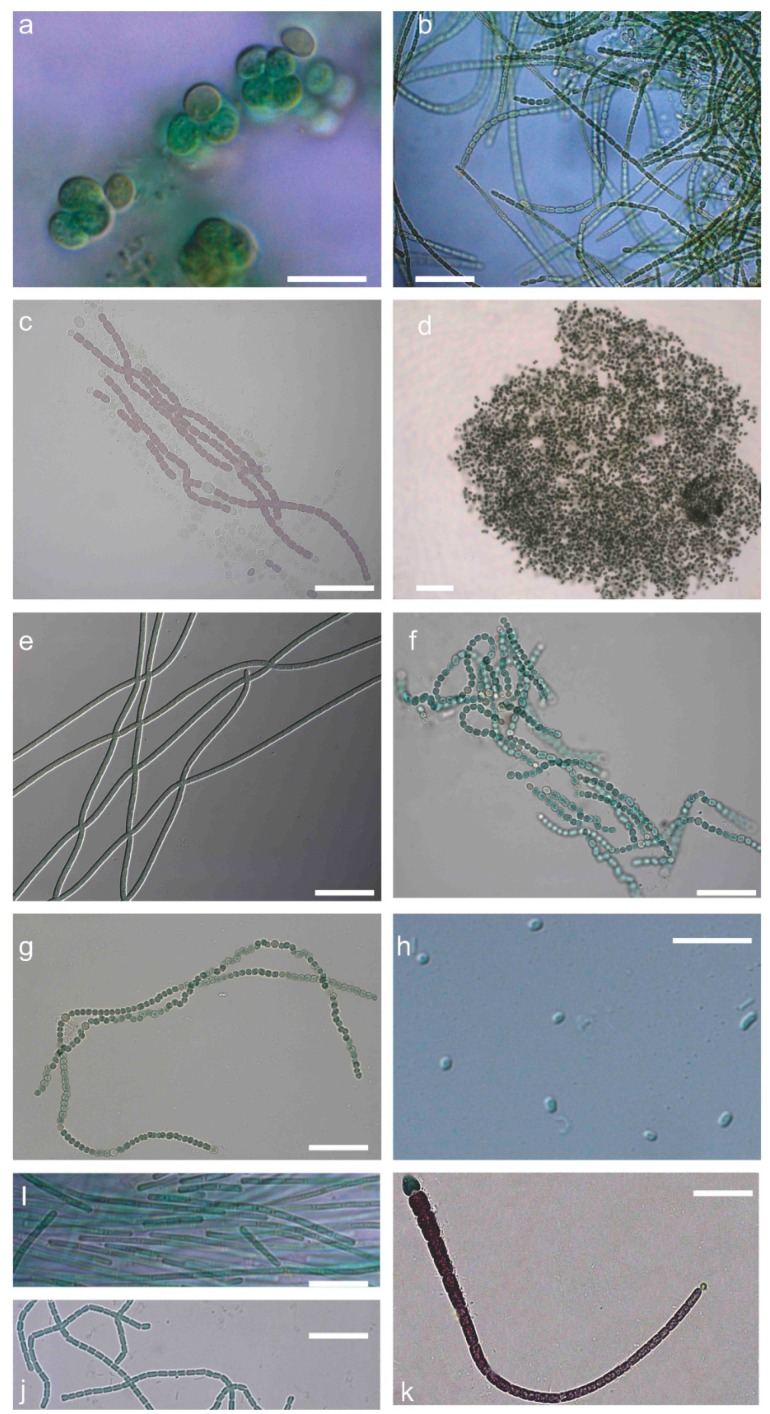
Microphotographs of strains representing 11 genera of cyanobacteria isolated from freshwaters of Greece. (**a**) *Chlorogloeopsis fritschii* TAU-MAC 0599; (**b**) *Desmonostoc muscorum* TAU-MAC 0699; (**c**) *Nostoc elgonense* TAU-MAC 0299; (**d**) *Microcystis flos-aquae* TAU-MAC 1410; (**e**) *Jaaginema* sp. TAU-MAC 0210; (**f**) *Trichormus variabilis* TAU-MAC 2510; (**g**) *Nostoc oryzae* TAU-MAC 2710; (**h**) *Synechococcus* cf. *nidulans* TAU-MAC 3010; (**i**) *Limnothrix redekei* TAU-MAC 0310; (**j**) *Nodosilinea* sp. TAU-MAC 0104; (**k**) *Calothrix epiphytica* TAU-MAC 0399. Scale bar = 20 μm.

According to the combined morphology, morphometric characters (Table S1, Supplementary Materials) and the phylogeny based on 16S–23S ribosomal RNA (rRNA) and *cpcBA*–internal genetic spacer (IGS) regions (Figure 1), our strains were classified into 11 genera and 17 taxa belonging to Chroococcales, Synechococcales, and Nostocales (Table 1). Five *Microcystis*, three *Jaaginema*, two *Nostoc*, one *Nodosilinea*, and one *Synechococcus* strains were identified up to the genus level, whereas the rest were identified up to the species level. *Microcystis* strains were placed in three different subclades; all strains morphologically identified as *M. flos-aquae* (Figure 2d) were placed in the same subclade (Figure 1). *Synechococcus* strains (Figure 2h) formed two subclades with other strains identified as *Cyanobium* and *Aphanothece* (Figure 1). *Limnothrix* and *Jaaginema* strains were placed in the same subclade and shared morphological characters (Figure 1, Table S1 and Figure S1, Supplementary Materials); *L. redekei* Thessaloniki Aristotle University Microalgae and Cyanobacteria (TAU-MAC) 0310 differed only in having both polar and a small central aerotope (Figure 2e,i). Strain TAU-MAC 0104 was clearly placed within *Nodosilinea* although it shared morphological characters with *Pseudanabaena*, and no nodules were observed under culture conditions (Figure 2j), even when cultured in 4 °C in the dark. Within Nostocales, *Chlorogloeopsis fritschii* TAU-MAC 0599 and *Calothrix epiphytica* TAU-MAC 0399 formed two distinct subclades with only one other strain in each subclade (Figure 1) and were identified by their morphology (Table S1, Supplementary Materials; Figure 2a,k). *Nostoc oryzae* TAU-MAC 2610 and 2710 formed a separate subclade with other *N. oryzae* strains; outside this clade was *Trichormus variabilis* TAU-MAC 2510 (Figure 1), identified by the formation of vegetative cells within a filament, slightly compressed in the middle and between two cells (Figure 2f).

### 2.2. Cyanotoxins

None of the strains were found to produce nodularin (NOD), cylindrospermopsin (CYN), or anatoxin-a (ATX-a). Nine strains, classified to *Microcystis flos-aquae*, *Microcystis* sp., and *Nostoc oryzae*, were found to produce 10 variants of microcystins (MCs) (Table 1). *Microcystis* strains produced predominantly MC-YR, MC-LR, [D-Asp^3^] MC-LR, and MC-HilR in total concentrations ranging from 833–3681 μg∙g^−1^ dry weight (dw); *Nostoc oryzae* TAU-MAC 2710 produced only MC-YR and traces of [D-Asp^3^] MC-LR and MC-LR with a total MC content up to 1.6 μg∙g^−1^ dw. MC-YR, MC-LR, and [D-Asp^3^]MC-LR were the predominant MCs in most of *Microcystis* strains with percentages of the total MC content ranging from 37 to 54%, from 22 to 38%, and from 1% to 14%, respectively. MC-HilR was also identified in those samples, its percentage of the total MC ranging from 2 to 4%, whereas MC-LA, MC-LY, and MC-LW were also present in minimal amounts. MC-RR, MC-WR, and [D-Asp^3^] MC-RR were only found in *Microcystis* sp. TAU-MAC 2310 strain with percentages of the total MC content to 30%, 4.5% and 1%, respectively. MC-LF was found only in *Microcystis* sp. TAU-MAC 2410 in trace amounts. No strain was found to produce MC-HtyR.

### 2.3. Antibacterial Activity

**Figure 3:**
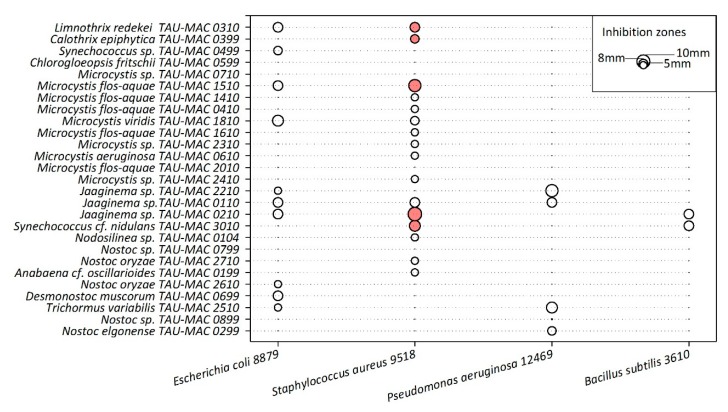
Inhibition zones produced by methanolic extracts of the studied cyanobacteria strains against four heterotrophic bacteria. Filled and empty circles represent clear and semi-transparent inhibition zones, respectively.

Twenty-two strains showed inhibition on the growth of the four heterotrophic bacteria tested (Figure 3); clear inhibition zones, the largest of which was 1.1 cm, were observed for five cyanobacterial extracts (*Microcystis flos-aquae* TAU-MAC 1510, *Synechococcus* cf. *nidulans* TAU-MAC 3010, *Jaaginema* sp. TAU-MAC 0210, *Limnothrix redekei* TAU-MAC 0310, and *Calothrix epiphytica* TAU-MAC 0399) only against *Staphylococcus aureus* 9518 (Figure 3). The inhibition zones in the rest of the heterotrophic bacteria were semi-transparent. Titration experiments showed that only *Synechococcus* cf. *nidulans* TAU-MAC 3010 completely inhibited the growth of *Staphylococcus aureus*; the rest of the strains showed moderate (*Microcystis flos-aquae* TAU-MAC 1510, *Jaaginema* sp. TAU-MAC 0210, *Limnothrix redekei* TAU-MAC 0310) or no (*Calothrix epiphytica* TAU-MAC 0399) inhibition (Figure S2, Supplementary Materials).

### 2.4. Effects on Cell Lines

**Figure 4:**
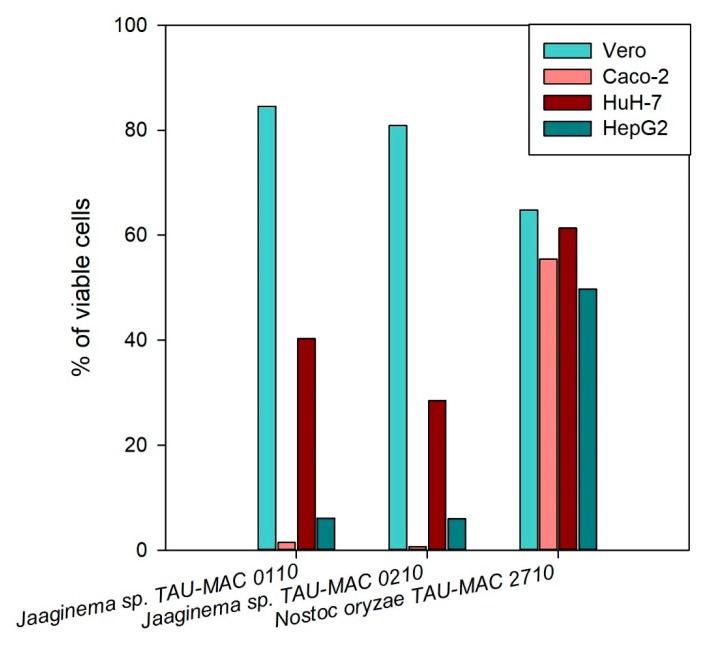
Cytotoxicity induced by methanolic extracts of *Jaaginema* sp. TAU-MAC 0110, *Jaaginema* sp. TAU-MAC 0210, and *Nostoc oryzae* TAU-MAC 2710 strains against Vero, Caco2, HuH-7, and HepG2 cell lines after 48 h of exposure.

The initial screening of the hydrophilic extracts was performed on the cancerous cell lines HepG2, HuH-7, and Caco2 in order to identify the strains with the strongest cytotoxic effects. Several MC-producing *Microcystis* strains had some impact on the viability of the cancerous cells (Figure S3, Supplementary Materials). The strains *Jaaginema* sp. TAU-MAC 0110 and 0210 presented the highest cytotoxicity effect in all cell lines; *Nostoc oryzae* TAU-MAC 2710 presented considerable cytotoxic activity in both HepG2 and HuH-7 cell lines; however, in the Caco2 cell line, the cytotoxicity was not at the same extent (Figure S3, Supplementary Materials). The methanolic extracts of these three strains were tested again on all cell lines: *Jaaginema* sp. TAU-MAC 0110 and 0210 presented high cytotoxic activity in all carcinoma cells lines but not in the Vero cells; *Nostoc oryzae* TAU-MAC 2710 presented only moderate cytotoxic activity in all cell lines (Figure 4). The same results were obtained when the experiment was repeated for the strains *Jaaginema* sp. TAU-MAC 0110 and 0210 that showed cytotoxicity in Caco2 and HepG2 cells, already in 24-h exposure in both 1:10 and 1:50 dilutions; no cytotoxic effect was observed this time from the *Nostoc oryzae* TAU-MAC 2710 strain (Figure S4, Supplementary Materials). In all experiments, HuH-7 cells were less susceptible compared to Caco2 and HepG2 cells.

**Figure 5:**
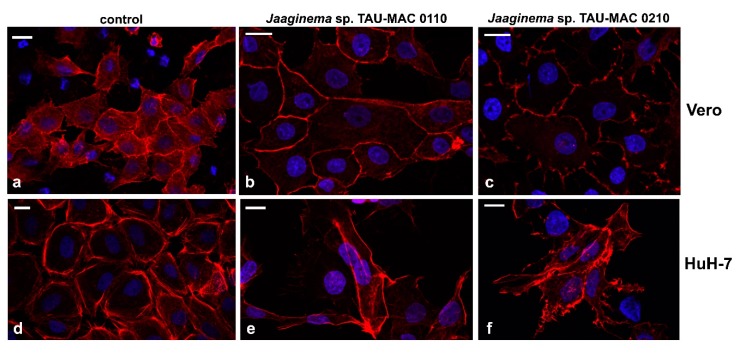
Actin and chromatin in Vero (**a**–**c**) and HuH-7 (**d**–**f**) cells after 48 h of exposure to *Jaaginema* sp. TAU-MAC 0110 (**b**,**e**) and *Jaaginema* TAU-MAC 0210 (**c**,**f**) extracts. Uninfluenced cells: a,d. Red, phalloidin–tetramethylrhodamine B isothiocyanate (TRITC) stained actin filaments; blue, chromatin visualized with 4′,6-diamidino-2-phenylindole (DAPI). Scale bar = 20 μm.

In order to further assess the cytotoxic effects, we observed Vero and HuH-7 cell lines exposed to strains *Jaaginema* sp. TAU-MAC 0110 and 0210 strain methanolic extracts after staining with phalloidin and 4′,6-diamidino-2-phenylindole (DAPI) (Figure 5). Vero cells appeared to be discolored (Figure 5b,c) compared to control cells (Figure 5a), suggesting a shift of actin from filamentous to spherical. Moreover, the *Jaaginema* sp. TAU-MAC 0210 extract caused some shrinking and the formation of filopodia-like membrane projections. The effects on actin network were more intense in HuH-7 cells: the cells were irregularly shaped without complying with a particular space arrangement, whereas filopodia-like membrane projections were more intense (Figure 5e). In the most affected HuH-7 cells, filopodia extended over a long length, were strongly branched, and the actin filaments exhibited irregular distribution (Figure 5f). The chromatin inside the nuclei appeared intact in all cases (Figure 5).

**Figure 6:**
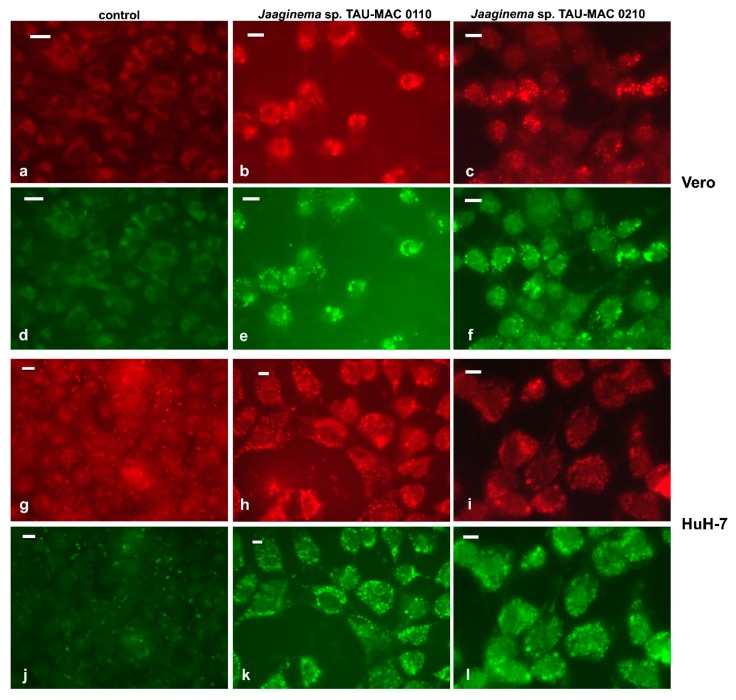
Fluorescently stained (Nile Red) lipid droplets in Vero (**a**–**f**) and HuH-7 (**g**–**l**) cells. Staining was done on fixed cells after 48 h of exposure with the extracts of *Jaaginema* sp. TAU-MAC 0110 (**b**,**e**,**h**,**k**) and *Jaaginema* sp. TAU-MAC 0210 (**c**,**f**,**i**,**l**) strains. Uninfluenced cells: **a**,**d**,**g**,j; green, Alexa Fluor 488 (**a**–**c** and **g**–**i**); red, Alexa Fluor 546 (**d**–**f** and **j**–**l**). Scale bar = 20 μm.

After a 48-h exposure to *Jaaginema* TAU-MAC 0110 and 0210 strains, cells of the Caco2, HepG2, and HuH-7 lines had their cytoplasm filled with small spherical structures (data not shown). These structures stained positively with the Nile Red lipophilic fluorescent dye and, therefore, were identified as lipid droplets (Figure 6). In Vero cells exposed for 48 h in both extracts, lipid droplets, scattered throughout the cytoplasm, with a slightly more pronounced distribution in the periphery, were distinct (Figure 6b,c,e,f). A similar emission pattern was observed in both red and green fluorescence, suggesting that lipid droplets comprised both polar and nonpolar lipids, considering the chemical properties of the Nile Red molecule. In the HuH-7 cell line, the effect was significantly more pronounced than in Vero cells; the lipid droplets in this case appeared numerous with almost uniform distribution throughout the cytoplasm (Figure 6h,i,k,l).

## 3. Discussion

The polyphasic taxonomy applied to the strains of this study revealed taxa known to be part of the bloom-forming communities in Greece, such as *Microcystis* and *Limnothrix* [23,24,25] and others, such as *Chlorogloeopsis fritschii*, *Desmonostoc muscorum*, *Nostoc elgonense*, *and Nodosilinea* sp., which were reported for the first time in Greek freshwaters [21].

In the present study, all 16–23S rRNA and *cpcBA*–IGS sequences of *Microcystis* strains, regardless of their morphospecies, were very similar, with sequence similarity ranging from 98–100%, corroborating the placement of all *Microcystis* species in a single branch [26,27,28,29]. The incongruence between the morphological and molecular diversity of *Microcystis* was repeatedly reported [27,28,30] and may be due to the fact that the morphological characteristics used to classify the various species of the genus are inconsistent with each other, while others may be lost after long-term cultivation of cyanobacteria of this genus [27,31]. In this study, only three of the 12 *Microcystis* strains were shown to maintain the aerotopes in their cells after long-term cultivation, and nine shifted from colonial to unicellular, exhibiting a striking phenotypic plasticity; this kind of plasticity is one of the features considered to enable *Microcystis* to dominate phytoplankton in eutrophic lakes [32].

The strain TAU-MAC 0104 was classified into the genus *Nodosilinea*, firstly described by Perkerson et al. [33], who separated *Leptolyngbya* strains able to form condensed structures from vegetative cells, described as nodules, under conditions of diminished illumination, into *Nodosilinea*. In the case of strain TAU-MAC 0104, the formation of nodules was not observed, neither in the initial isolation stages, nor under low-light conditions; nevertheless, phylogenetic analysis clearly placed it within *Nodosilinea*, defined by 16S rRNA and the well-conserved secondary structure of the 16S–23S internal transcribed spacer (ITS) rRNA [33]. *Jaaginema* TAU-MAC 0110, 0210, and 2210 and *Limnothrix* 0310 strains were grouped together with other *Limnothrix* strains indicating the current taxonomic uncertainty of *Jaaginema* [34]. The genus *Jaaginema* includes mainly benthic members of the genus *Oscillatoria*, which is known to be polyphyletic and is distributed within different branches in phylogenetic trees, not always consistent with its morphological characteristics [29,35,36]. This was also observed for *Jaaginema*; strains such as *J. neglecta* were placed close to *Leptolyngbya* [37], whereas in this work *Jaaginema* strain 0210 showed a 99% sequence similarity with *Limnothrix planctonica* CHAB763 strain. There is currently insufficient information to elucidate the phylogenetic position of *Jaaginema* [34] and, therefore, further molecular data are needed to clarify its classification. The great similarity (99–100%) of our three *Jaaginema* strains with the genus *Limnothrix* and the subclades formed when those two genera were compared suggests that the relationships between the two genera should be redefined.

Our phylogenetic analysis of Nostocales strains, showed the heterogeneity and polyphyly of *Nostoc* [28,38] with greater resolution in phylogeny, as a result of the emergence of new genera (e.g., *Desmonostoc*) and the use of different markers such as *cpcBA*–IGS. In our study, *Nostoc* strains formed a clade only with other *Trichormus* strains, which is in conflict with the position of *Nostoc* sequences in cyanobacteria phylogeny [38,39,40]. The rare nostocalean species *Chlorogloeopsis fritschii* was recorded mainly at thermal springs and wet stones [41,42] and, to the best of our knowledge, *Chlorogloeopsis fritschii* TAU-MAC 0599 is the first record of this species in a temperate lake. *Chlorogloeopsis fritschii* TAU-MAC 0599 was initially assigned to *Chroococcus* genus due to the absence of heterocytes and its occurrence as small aggregates [22] reminiscent of *Chroococcus* [41]. Similarly, no heterocytes were mentioned in the original description of *Chlorogloea fritschii* (*Chlorogloeopsis fritschii*) by Mitra in 1950 [41]; therefore, the author of the species classified it into the chroococcalean family Entophysalidaceae.

Microcystins were found in nearly 70% of the *Microcystis* strains examined in this study, thus providing further evidence that cyanobacteria blooms in Greece, often dominated by *Microcystis* spp., are highly likely to contain microcystins [23,24,25,43]. All MC-producing *Microcystis* strains from the present study were isolated from Pamvotis Lake, in northwest (NW) Greece [22]. Microcystins MC-RR, MC-LR, and MC-YR are the main toxin constituents of the Greek bloom samples, as well as in blooms from other regions of southern and central Europe countries, like Portugal, France, Germany, Poland, and in other parts of the world including Japan and South Korea [44]. Interestingly, previous studies [23] showed that MC-RR was absent in Lake Pamvotis, indicating that the dominant *Microcystis* spp. produce mainly MC-LR. Our results suggest that, in Lake Pamvotis, strains capable of producing both MC-LR and MC-RR occur, along with their desmethylated congeners, and this is corroborated in a recent study [25]. Data from *Microcystis* strains from other regions of the world show that *Microcystis* produce lower (France, [45]; Singapore, [46]); higher (Portugal, [47]), or similar (Spain, [48]) MC concentrations.

The genus *Nostoc* is common in both terrestrial and aquatic habitats, typically growing on sediments or stones in the littoral or in running water [49]. Recently, increasing evidence on the worldwide abundance of *Nostoc* spp. as a MC-producing organism was reported [50,51,52]. Our strain *Nostoc oryzae* TAU-MAC 2710 produced MC-YR and despite the fact that the MC content was very low; to the best of our knowledge, this is the first report of *Nostoc oryzae* producing MCs. Considering that *Nostoc oryzae* is a critical inhabitant of rice fields [53], the range of habitats in which MC production was documented is broadened according to this observation, with possible implications of MC transfer through the food web. MCs may transfer to rice farms through freshwater used for agricultural irrigation. In Lake Taihu (China), the water used in rice fields was found to contain a wide range of MCs (0.22–3.19 μg∙kg^−1^) [54].

Antibacterial activities of extracts from representatives of Nostocales, Chroococcales, and Synechococcales were observed against the Gram-positive bacterium *Staphylococcus aureus* (Figure 3). Similarly, previous studies testing the effects of cyanobacterial extracts found inhibition effects only against Gram-positive bacteria [55,56]. This could be attributed to the resistance of Gram-negative bacteria to toxic agents due to their protective role of the lipopolysaccharide layer of the outer membrane [57]. Antimicrobial activities of cyanobacteria were mostly reported from filamentous strains [55,58,59], and relatively little were found from coccoid morphs. Martins et al. [56] assessed the biological activities of marine *Synechocystis* and *Synechococcus* strains and their results suggested that these genera could be a source of antibiotic compounds. In accordance with these results, our *Synechococcus* cf. *nidulans* TAU-MAC 3010 showed significant activity. Although many antimicrobial activities were reported from cyanobacterial strains, only few molecules responsible for these activities were identified. Further investigation is required in order to identify the secondary metabolites responsible for these antimicrobial activities.

Regarding cytotoxic activity, aquatic extracts had less activity than the methanolic ones, as it seems that methanol is more efficient for extracting compounds with higher cytotoxic activity. This fact was already described in previous works [3,60,61], where hydrophilic extracts were used as an initial screening, following by a methanolic bioactivity assay. In the last decade, strains representing several cyanobacteria genera (*Nodosilinea*, *Leptolyngbya*, *Pseudanabaena*, *Romeria*, *Oscillatoria*, *Cyanobium*, *Synechocystis*, *Synechococcus* etc.) isolated from diverse habitats (marine, estuarine, soil etc.) were tested against cancer cell lines and found to have strong cytotoxicity effects [20,56]. In this study, the HuH-7 cell line exposure to *Jaaginema* TAU-MAC 0110 and 0210 strains caused pronounced changes in the actin network, which is responsible for the shape of the cells and constitutes the main supportive structure of filopodia and lamellipodia [62]. The most characteristic changes we observed were cell shrinkage and formation of irregular membrane filopodia-like projections, which suggest a direct effect on actin; toxins that affect the actin network trigger depolymerization of actin filaments and subsequent reassembly, either in the perinuclear space or at the periphery of the cell, resulting in the formation of membrane projections and filopodia [63]. Displacement of the actin cortex in the perinuclear space is a common phenomenon in detached cells [64], while the loss of normal adhesion to the extracellular matrix was associated with apoptosis [65]. The pronounced cytotoxicity pattern at the intracellular level is consistent with the low survival rates we observed in the 3-(4,5-dimethylthiazol-2-yl)-2,5-diphenyl tetrazolium bromide (MTT) assay. Elongated membrane projections, despite not including organelles, were also observed in cases of cytotoxicity of cyanobacterial extracts that caused apoptotic death of unusual morphological features in rat hepatocytes [66]. In similar studies, marine *Synechocystis* and *Leptolyngbya* strains were found to induce cytotoxic effects in the hepatocellular carcinoma HepG2 cell line [20]. Further, *Nostoc* and *Tolypothrix* strains from soil habitats were also tested against the HepG2 cell line and 90% of crude cyanobacterial extracts were found to induce cytotoxic effects [67]. In all the above-mentioned cases, the compounds responsible for cytotoxicity, as well as the mechanisms of action, are not known.

The formation of numerous lipid droplets we observed in Vero and HuH-7 cell lines, after a 48-h exposure to *Jaaginema* TAU-MAC 0110 and 0210 extracts, was associated with cytotoxicity models in several cases (e.g., Reference [68]). Lipid droplets are cytoplasmic organelles, bound by a phospholipid monolayer, which are found in the majority of cell types; they are the major cellular organelles for the storage of neutral lipids, such as triacylglycerols and sterols [69]. In addition, they contain enzymes involved in their metabolism, such as coenzyme A and various lipases [70]. Exposure of THP-1 human leukemia cells to diesel exhaust particles induced the accumulation of lipid droplets after 24 h; however, the phenomenon was not associated with oxidative stress [68]. In contrast, lipid droplets in mouse PC12 cancer cells treated with CdT are a major cause of oxidative stress [71]. One of the results of oxidative stress is the injury of the mitochondrial membrane, which disrupts the β-oxidation pathway of fatty acids and leads to the storage of triglycerides, resulting in a significant increase of lipid droplets [72]. Considering the low viability rates of the HuH-7 cell line in the MTT assay, the formation of lipid droplets suggests mitochondrial damage.

In this study, non-toxic strains had more acute effects, whilst strains with less bioactivity synthesized only MCs amongst the targeted cyanotoxins. MCs were already described to pose antimicrobial activity in non-resistant microbes and induce apoptosis in human cancer cell lines [73,74]. Compounds related with higher bioactivity in literature, however, represent more complex chemical classes [75,76,77], as well as compounds that are not yet characterized [78,79,80]. Our findings suggest that the compounds responsible for the bioactivity presented by the non-toxic TAU-MAC strains are yet unknown or not identified.

Metabolomic intraspecies variation is a phenomenon well documented in planktonic and freshwater cyanobacteria [9,70]. The reason why certain strains are not able to synthesize specific compounds is unclear, but it was speculated that mutations within the compound gene cluster might have occurred either during cultivation or under natural conditions [81,82,83]. In our study, the strains *Jaaginema* TAU-MAC 0110, 0210, and 2210 share highly similar 16S rRNA sequences; however, different activities are depicted amongst those three strains. The 16S rRNA sequence similarity is also high (>99%) between *Nostoc oryzae* TAU-MAC 2610 and 2710, whilst only 2710 possesses the capability of MC biosynthesis. Regarding the *Microcystis* genus, research focuses on the presence of both toxic and non-toxic *Microcystis* communities in natural populations [84,85]. Based on our results, we could highlight that only one *Microcystis flos-aquae* strain (TAU-MAC 1510) exhibited antimicrobial activity, even though similarity above 99% was shared amongst all *Microcystis flos-aquae* TAU-MAC strains, based on 16S rRNA sequences.

## 4. Conclusions

Our multidisciplinary assessment on freshwater cyanobacteria strains broadened our knowledge on some species habitats and revealed their metabolic diversity. Twenty-nine cyanobacterial strains were assigned to three orders representing 11 genera. There is good agreement between 16S rRNA–*cpcBA*–IGS characterization and morphological features, except for the *Jaaginema*–*Limnothrix* group which appears intermixed and needs further elucidation. We showed for the first time that *Chlorogloeopsis fritschii* may occur in freshwater temperate habitats, and that *Nostoc oryzae* isolated from rice fields is capable of producing MCs, amongst the most dominant MCs produced by *Microcystis* strains. Strains with very high or identical 16S rRNA gene sequences displayed different antibacterial and cytotoxic activities. Methanol extracts from *Synechococcus* cf. *nidulans* showed the most potent antibacterial activity, whereas *Jaaginema* sp. strains exhibited potent cytotoxic activities against human colorectal adenocarcinoma and hepatocellular carcinoma cells. *Jaaginema* TAU-MAC 0110 and 0210 strains caused pronounced changes in the actin network and triggered the formation of numerous lipid droplets in hepatocellular carcinoma and green monkey kidney cells, suggesting oxidative stress and/or mitochondrial damage leading to apoptosis.

## 5. Materials and Methods

### 5.1. Cyanobacterial Strains and Culture

Twenty-nine strains of cyanobacteria of the Thessaloniki Aristotle University Microalgae and Cyanobacteria (TAU-MAC) culture collection [86] were used in this study. The strains were isolated from Greek freshwaters between 1999 and 2010 and were classified to the genera *Anabaena*, *Dolichospermum*, *Calothrix*, *Chroococcus*, *Jaaginema*, *Limnothrix*, *Microcystis*, *Pseudanabaena*, and *Synechococcus* based on their morphology [22]. The cultures were grown in BG11 medium with or without (for the nitrogen-fixing strains) the addition of inorganic nitrogen [87]. Cultures were maintained at 20 ± 1 °C or 25 ± 1 °C (for *Microcystis* strains) with a light intensity of 25 μmol∙m^−2^∙s^−1^ and with a light/dark cycle of 12:12 h.

### 5.2. Polyphasic Taxonomy

Morphological examination of cyanobacteria isolates was performed using a Zeiss Axio imager z2 microscope. The strains were identified to the species or genus level according to Komárek and Anagnostidis [88] and Komárek [49]. Microphotographs were taken with an Axio Cam MRc5 digital camera (Carl Zeiss, Germany). Mean cell or filament dimension was calculated after measuring the dimensions of at least 50 individuals (cells or filaments) of each strain.

The molecular phylogeny of the strains was assessed by the 16S rRNA gene, the 16S–23S rRNA internal transcribed spacer (ITS), and the phycocyanin operon and its internal genetic spacer (*cpcBA*–IGS). PCR was carried out using the primer pairs shown in Table S3 (Supplementary Materials) and PCR conditions described in the references therein. PCR was carried out using an Eppendorf MasterCycler Pro (Eppendorf). PCR products were separated by 1.5% (*w*/*v*) agarose gel electrophoresis in 1× TAE buffer. The gels were stained with Midori Green Advanced (NIPPON Genetics Europe GmbH) and photographed under ultraviolet (UV) transillumination. Sequence data were obtained by capillary electrophoresis (GENEWIZ, Takeley, UK). The obtained nucleotide sequences were edited with Unipro UGENE 1.29.0. Nucleotide sequences were deposited in GenBank database of the National Center for Biotechnology Information (NCBI) (Table S2, Supplementary Materials).

All new sequences were Basic Local Alignment Search Tool (BLAST)ed and the closest relative(s) for each sequence were included in the phylogenetic trees. For the phylogenetic analyses, we selected sequences (>600 bp for *cpcBA*–IGS and >1500 bp for 16–23S rRNA), deposited in GenBank in order to examine phylogenetic position of our strains (Table S4, Supplementary Materials). Phylogenetic analysis was conducted with Mega (V7.0) software [89]. Complete deletion option was selected for all missing data and gaps. The maximum-likelihood (ML) method was used for the construction of a consensus phylogenetic tree and Tamura 3-parameter + G as the best-fitting evolutionary model. Bootstrap replicates (*n* = 1000) were performed. Phylogeny was also inferred with the Bayesian inference (BI) phylogenetic approach with MrBayes (V3.2.6) software [90]. The evolutionary model used was selected by applying PAUP* (V5.0) [91]. The general time-reversible model (GTR) with gamma distribution of rates and a proportion of invariable sites was selected. Bayesian analysis consisted of two independent Markov chain Monte Carlo runs, performed by four differentially heated chains of 10 × 10^6^ generations, and trees were sampled from the chain every 1000 generations. All phylogenetic trees were visualized using the FigTree (V1.4.3) software [92].

### 5.3. Cyanotoxin Analysis Using LC–MS/MS

Cyanotoxins (12 MCs, CYN, NOD, and ATX-a) were determined in lyophilized culture material (40–200 mg). Biomass was suspended in 1.5 mL of 75% (*v*/*v*) aqueous methanol and sonicated for 15 min. After sonication, the mixture was centrifuged for 10 min at 4000 rpm and the supernatant was collected. The procedure above was repeated two times, following the addition of 1.5 mL 75% (*v*/*v*) aqueous methanol and 1.5 mL of butanol. The resulting solutions were pooled together, evaporated, and the residue was dissolved in 1 mL of 5% (*v*/*v*) aqueous methanol and filtered (0.2 μm) before LC–MS/MS analysis. Chemical analysis was carried out on a Finnigan TSQ Quantum Discovery Max triple-stage quadrupole mass spectrometer (Thermo Fischer Scientific, Waltham, MA, USA), equipped with electrospray ionization (ESI) source and a Finnigan Surveyor AS autosampler (Thermo Fischer Scientific). Detection was performed in multiple reaction monitoring (MRM) mode. Xcalibur software 2.1 SP 1160 was used to control the mass spectrometer and for data acquisition. The determination of CYN, ATX-a, NOD, and MCs ([D-Asp^3^]MC-RR, MC-RR, MC-YR, MC-HtyR, [D-Asp^3^]MC-LR, MC-LR, MC-HilR, MC-WR, MC-LA, MC-LY, MC-LW, MC-LF) was carried out according to the LC-MS/MS method described by Zervou et al. [93]. The method limits of detection (LOD) for target cyanotoxins were 0.1 μg∙g^−1^ dw for CYN, 0.3 μg∙g^−1^ dw for ATX-a, 0.2 μg∙g^−1^ dw for NOD, and ranged from 0.1 to 0.7 μg∙g^−1^ dw for MCs.

### 5.4. Extract Preparation for Assays

Cyanobacteria cells were harvested at the exponential growth phase (between 30 and 45 days of growth) by centrifugation of the whole liquid culture (250 mL) and were freeze-dried. The lyophilized biomass (6–20 mg dry weight) was dissolved in 4 or 8 mL of double-distilled water and sonicated for 8∙10 min. The same extraction procedure was repeated with 75% (*v*/*v*) MeOH according to the protocol described in Gkelis et al. [24], as the use of aqueous methanol is more efficient in extraction a broader range of compounds [3,60,61]. In this protocol, MeOH is evaporated in the final step of extraction and the residue is dissolved in 1 mL of Milli-Q water. Extracts were filtered through a 0.45-μm sterile syringe filter (Whatman, GE Healthcare Life Sciences, Chicago, IL, USA).

### 5.5. Heterotrophic Bacteria and Antibacterial Assays

Four heterotrophic bacteria strains, *Escherichia coli* 8879, *Pseudomonas aeruginosa* 12469, *Bacillus subtilis* 3610, and *Staphylococcus aureus* 9518, were used for antibacterial assays. The cultures were grown in Nutrient Broth [94] except for *Staphylococcus aureus* 9518, which was grown on *Staphylococcus* medium No. 110 (Thermo Scientific). The four bacterial strains were spread on Nutrient Broth Agar (1.5% *w*/*v*) plates and 0.5-cm-diameter filter paper discs impregnated with the extracts were placed on the plates. After 48 h of incubation at 37 °C, the inhibition zones were measured for every cyanobacterial extract. Double-distilled water (ddH_2_O) was used as negative control. For extracts showing clear inhibition zones, the bacterial growth was evaluated by titration: 10-mL liquid cultures were incubated in 37 °C for 24 h; a 1-mL aliquot was centrifuged and the pellet was resuspended in 250 μL 2× *Staphylococcus* medium 110, 250 μL 1× *Staphylococcus* medium 110, and 250 μL of the cyanobacterial extract (or 250 μL ddH_2_O for negative control). Colony-forming units (cfu)∙mL^−1^ were calculated at 0, 3, 8 and 24 h; cfu were calculated from 100 μL of the liquid culture, after serial dilutions in growth medium up to 10^−6^ *v*/*v*, plating of the last three dilutions (10^−4^ *v*/*v*, 10^−5^ *v*/*v*, 10^−6^ *v*/*v*) on agar plates, and counting the colonies after 24 h of incubation at 37 °C.

### 5.6. Cell Lines and Cytotoxicity Assay

Four cell lines were employed in this study: human colorectal adenocarcinoma (CaCo2), hepatocellular carcinoma (HepG2), hepatocellular carcinoma (HuH-7), and African green monkey kidney cells (Vero). These cell lines are suitable to study the toxic effects of cyanotoxins, as they hold the capability to express transmembrane solute carriers transport family OAPT (organic anion polypeptide transporters) [95,96]. Cell lines were cultivated in Dulbecco’s modified Eagle medium (DMEM) except Vero cells which were grown in minimum essential medium (MEM). DMEM was supplemented with 10% fetal bovine serum (FBS), l-glutamine (2 mM), and Penicillin/Streptomycin (10 U∙mL^−1^). MEM medium was supplemented with 10% FBS, l-glutamine (2 mM), 1% (*v*/*v*) non-essential amino acids (0.1 mM), Penicillin/Streptomycin (10 U∙mL^−1^), sodium pyruvate (1 M), and sodium bicarbonate (1500 mg∙mL^−1^). The cell lines were incubated in an atmosphere of 5% *v*/*v* CO_2_ and at 37 °C. Cells were grown attached to the surface tending to form monolayers. Cell line re-culturing was carried out every 2–3 days when the cells covered 70–80% of the flask attachment surface [97].

The toxic potential of the cyanobacterial extracts was estimated using the MTT (3-(4,5-dimethylthiazol-2-yl)-2,5-diphenyl tetrazolium bromide) assay, based on the reduction of the MTT performed only in viable cells with active metabolism [98]. Cells were initially seeded in 48-well plates. After 24 h, cell adhesion was completed and the cells were exposed to aqueous extracts for 24 h in 1:10 (*v*/*v*) dilution. For strains showing activity against cell lines, the assay was repeated with methanolic extracts for all cell lines in 1:10 (*v*/*v*) dilution exposed for 48 h. The experiment was repeated in 1:10 and 1:50 (*v*/*v*) dilutions for the cell lines CaCo2 and HepG2 exposed for 24 h and 48 h. After exposure, cells were incubated for 2 h, at 37 °C, with a 5 mg∙mL^−1^ solution of MTT in phosphate-buffered saline (PBS) (NaCl (8 g∙L^−1^), KCl (0.8 g∙L^−1^), Na_2_HPO_4_ (1.44 g∙L^−1^), KH_2_PO_4_ (0.22 g∙L^−1^), pH 7.4). The colored formazan salts formed were dissolved in isopropanol and the absorbance was read at 570 nm (optical density, OD_570_). Negative controls for each assay consisted of cells grown in fresh culture medium. The experiments were run in triplicate and mean values are given. Percentages of viable cells were estimated as the percentage of the OD_570_ compared to the OD_570_ of the negative control, which was considered as 100% viable.

We further examined the cytotoxic effects of the extracts on carcinoma cell lines, using phalloidin, DAPI (4′,6-diamidino-2-phenylindole), and Nile Red stainings. After 24-h treatment with extracts, as described earlier, the extract was removed and the coverslips were washed gently once with PBS pH 7.4 at 37 °C. Cells were fixed with freshly prepared 3.7% *v*/*v* paraformaldehyde (PFA) in PBS buffer for 5 min at room temperature. After subsequent washing twice with PBS, 0.5% triton X-100 was added and the cells were again washed twice with PBS and stained with 0.4 μΜ diluted phalloidin–TRITC (tetramethylrhodamine B isothiocyanate, P5282), which specifically stains the actin filaments (see Reference [99]). Following incubation for 3 h in dark, the samples were washed twice with PBS and once with distilled water to remove PBS, and then counterstained with DAPI (Braunschweig Chemie) containing antifade medium (90% glycerol, 2% *v*/*v* DABCO, 20 mM Tris–HCl, 0.02% *w*/*v* sodium azide, pH 8.0) for nuclear staining. For Nile Red staining, the stock solution (250 mg∙mL^−1^) was diluted 1:1000 *v*/*v* in PBS; stained cells were incubated for 15 min in dark and washed with 1 mL of PBS. The preparations were examined with a Nikon D-Eclipse C1 or a Zeiss LSM780 confocal laser scanning microscope (CLSM), with the appropriate filters for FITC, and micrographs were acquired with the manufacturer’s software.

## Figures and Tables

**Table 1:** Concentration of cyanotoxins in the strains studied as determined by LC–MS/MS analysis.

Strain	Toxin Concentration (μg∙g^−1^ Dry Weight)												
	[D-Asp^3^] MC-RR	MC-RR	MC-YR	MC-HtyR	[D-Asp^3^] MC-LR	MC-LR	MC-HilR	MC-WR	MC-LA	MC-LY	MC-LW	MC-LF	Total
*Microcystis aeruginosa* TAU-MAC 0610	-*	*-*	*-*	*-*	*-*	*-*	*-*	*-*	*-*	*-*	*-*	*-*	
*Microcystis flos-aquae* TAU-MAC 0410	*-*	*-*	1029.6	*-*	366.4	728.4	48.3	*-*	<LOQ	<LOQ	2.3	*-*	2175.0
*Microcystis flos-aquae* TAU-MAC 1410	*-*	*-*	1379.6	*-*	227.4	809.2	50.0	*-*	<LOQ	<LOQ	2.6	*-*	2468.8
*Microcystis flos-aquae* TAU-MAC 1510	*-*	*-*	704.7	*-*	293.8	489.0	32.9	*-*	<LOQ	<LOQ	<LOQ	*-*	1520.4
*Microcystis flos-aquae* TAU-MAC 1610	*-*	*-*	*-*	*-*	*-*			*-*	*-*	*-*	*-*	*-*	
*Microcystis flos-aquae* TAU-MAC 2010	*-*	*-*	860.9	*-*	241.6	535.5	33.4	*-*	<LOQ	*tr.*	1.9	*-*	1673.3
*Microcystis viridis* TAU-MAC 1810	*-*	*-*	*-*	*-*	*-*	*-*	*-*	*-*	*-*	*-*	*-*	*-*	
*Microcystis* sp. TAU-MAC 0710	*-*	*-*	*-*	*-*	*-*	*-*	*-*	*-*	*-*	*-*	*-*	*-*	
*Microcystis* sp. TAU-MAC 1710	*-*	*-*	434.6	*-*	55.90	317.7	22.2	*-*	<LOQ	<LOQ	2.6	*-*	833.0
*Microcystis* sp. TAU-MAC 2110	*-*	*-*	1983.7	*-*	347.6	1257.1	79.7	*-*	1.2	2.9	8.7	*-*	3680.9
*Microcystis* sp. TAU-MAC 2310	11.6	360.6	445.7	-	10.7	269.8	54.0	54.6	-		*-*	*-*	1207.0
*Microcystis* sp. TAU-MAC 2410	*-*	*-*	1533.5	*-*	430.3	1105.8	64.3	*-*	<LOQ	1.8	4.3	<LOQ	3140.0
*Synechococcus* sp. TAU-MAC 0499	*-*	*-*	*-*	*-*	*-*	*-*	*-*	*-*	*-*	*-*	*-*	*-*	
*Synechococcus* cf. *nidulans* TAU-MAC 3010	*-*	*-*	*-*	*-*	*-*	*-*	*-*	*-*	*-*	*-*	*-*	*-*	
*Jaaginema* sp. TAU-MAC 0110	*-*	*-*	*-*	*-*	*-*	*-*	*-*	*-*	*-*	*-*	*-*	*-*	
*Jaaginema* sp. TAU-MAC 0210	*-*	*-*	*-*	*-*	*-*	*-*	*-*	*-*	*-*	*-*	*-*	*-*	
*Jaaginema* sp. TAU-MAC 2210	*-*	*-*	*-*	*-*	*-*	*-*	*-*	*-*	*-*	*-*	*-*	*-*	
*Limnothrix redekei* TAU-MAC 0310	*-*	*-*	*-*	*-*	*-*	*-*	*-*	*-*	*-*	*-*	*-*	*-*	
*Nodosilinea* sp. TAU-MAC 0104	*-*	*-*	*-*	*-*	*-*	*-*	*-*	*-*	*-*	*-*	*-*	*-*	
*Anabaena* cf. *oscillarioides* TAU-MAC 0199	*-*	*-*	*-*	*-*	*-*	*-*	*-*	*-*	*-*	*-*	*-*	*-*	
*Chlorogloeopsis fritschii* TAU-MAC 0599	*-*	*-*	*-*	*-*	*-*	*-*	*-*	*-*	*-*	*-*	*-*	*-*	
*Desmonostoc muscorum* TAU-MAC 0699	*-*	*-*	*-*	*-*	*-*	*-*	*-*	*-*	*-*	*-*	*-*	*-*	
*Nostoc elgonense* TAU-MAC 0299	*-*	*-*	*-*	*-*	*-*	*-*	*-*	*-*	*-*	*-*	*-*	*-*	
*Nostoc oryzae* TAU-MAC 2610	*-*	*-*	*-*	*-*	*-*	*-*	*-*	*-*	*-*	*-*	*-*	*-*	
*Nostoc oryzae* TAU-MAC 2710	*-*	*-*	1.6	*-*	<LOQ	<LOQ	*-*	*-*	*-*	*-*	*-*	*-*	1.6
*Nostoc* sp. TAU-MAC 0799	*-*	*-*	*-*	*-*	*-*	*-*	*-*	*-*	*-*	*-*	*-*	*-*	
*Nostoc* sp. TAU-MAC 0899	*-*	*-*	*-*	*-*	*-*	*-*	*-*	*-*	*-*	*-*	*-*	*-*	
*Trichormus variabilis* TAU-MAC 2510	*-*	*-*	*-*	*-*	*-*	*-*	*-*	*-*	*-*	*-*	*-*	*-*	
*Calothrix epiphytica* TAU-MAC 0399	*-*	*-*	*-*	*-*	*-*	*-*	*-*	*-*	*-*	*-*	*-*	*-*	

* not detected; <LOQ: below the limit of quantification, μg∙g^−1^ dry weight ([D-Asp^3^]MC-RR = 0.6, MC-RR = 0.3, MC-YR = 1.3, MC-HtyR = 2.0, [D-Asp^3^]MC-LR = 1.1 MC-LR = 1.3, MC-HilR = 1.9, MC-WR = 1.7, MC-LA = 0.9, MC-LY = 1.8, MC-LW = 1.3, MC-LF = 1.6).

## Supplementary Materials

The following are available online at https://www.mdpi.com/2072-6651/11/8/436/s1: Table S1: Morphometric characteristics of the studied strains; Table S2: GenBank accession numbers for TAU-MAC strains used in the phylogenetic analysis; Table S3: PCR primers used the phylogenetic analysis of cyanobacteria strains of TAU-MAC culture collection; Figure S1: Phylogenetic consensus tree for *Jaaginema* and *Limnothrix* spp. based on 16S–23S rRNA and *cpcBA*–IGS sequences of TAU-MAC strains, reconstructed using the maximum-likelihood (ML) analysis; Figure S2: Inhibition of *Staphylococcus aureus* 9518 growth by the five cyanobacteria strains that showed clear inhibition zones; Figure S3: Cytotoxicity induced by aquatic extracts of the studied strains against Caco2, Huh-7, and HepG2 carcinoma cell lines after 24-h exposure; Figure S4: Cytotoxicity induced by methanolic extracts of three cyanobacterial strains against Caco2 and HepG2 carcinoma cell lines after 24-h and 48-h exposure in 1:10 and 1:50 dilutions.
