# Supplementary material for: Diversity, Cyanotoxin Production, and Bioactivities of Cyanobacteria Isolated from Freshwaters of Greece
Source: Toxins (Basel). 2019 Jul 25;11(8):436. doi: 10.3390/toxins11080436 (PMC6723990; doi:10.3390/toxins11080436)
Supplement: Supplementary file 1 [file toxins-11-00436-s001.pdf]

Supplementary material: Diversity, Cyanotoxin Production, and Bioactivities of Cyanobacteria Isolated from Freshwaters of Greece

Spyros Gkelis, Manthos Panou, Despoina Konstantinou, Panagiotis Apostolidis[1], Antonia Kasampali, Sofia Papadimitriou, Dominiki Kati, Giorgia Maria Di Lorenzo, Stamatia Ioakeim, Sevasti-Kiriaki Zervou, Christophoros Christophoridis, Theodoros M. Triantis, Triantafyllos Kaloudis, Anastasia Hiskia and Minas Arsenakis

Table S1. Morphometric characteristics of the studied strains.

Strain	vegetative cell			heterocyte			akinete		
	length* (µm)	width (µm)	l:w	length (µm)	width (µm)	l:w	length (µm)	width (µm)	l:w
Microcystis aeruginosa TAU-MAC 0610	7.5 ± 0.9 [5.8–9.7]	7.2 ± 0.8 [5.7–9.3]	1.04 ± 0.01						
Microcystis flos-aquae TAU-MAC 0410	10.4 ± 1.0 [7.8–12.6]	9.0 ± 1.1 [6.9–12]	1.15 ± 0.2						
Microcystis flos-aquae TAU-MAC 1410	8.0 ± 0.9 [4.7–10.4]	8.0 ± 0.9 [5.7–10.7]	1 ± 0.2						
Microcystis flos-aquae TAU-MAC 1510	9.2 ± 1.2 [5.4–12.9]	7.9 ± 1.1 [5.1–10.3]	1.16 ± 0.1						
Microcystis flos-aquae TAU-MAC 1610	8.7 ± 1.1 [6.4–11.3]	7.9 ± 1.1 [5.4–11.8]	1.1 ± 0.01						
Microcystis flos-aquae TAU-MAC 2010	8.8 ± 1.2 [6.2–12.5]	7.9 ± 1.1 [5.5–11.8]	1.11 ± 0.05						
Microcystis viridis TAU-MAC 1810	8.3 ± 1.0 [5.8–11]	7.1 ± 1.0 [4.3–9.3]	1. 16 ± 0.1						
Microcystis sp. TAU-MAC 0710	7.3 ± 0.9 [5.3–10.2]	7.9 ± 0.7 [6.1–9.6]	0.9 ± 0.03						
Microcystis sp. TAU-MAC 1710	8.7 ± 0.9 [6.7–11.2]	7.5 ± 1.0 [5.5–10.3]	1.16 ± 0.2						
Microcystis sp. TAU-MAC 2110	8.2 ± 1.0 [6.2–11]	7.0 ± 0.9 [5.4–9.3]	1.17 ± 0.06						
Microcystis sp. TAU-MAC 2310	6.0 ± 1.0 [3–8.3]	5.5 ± 1.0 [3.3–8]	1.09 ± 0.03						
Microcystis sp. TAU-MAC 2410	9.2 ± 1.2 [5.9–12.5]	8.4 ± 1.4 [5.9–12.3]	1.16 ± 0.4						
Synechococcus sp. TAU-MAC 0499	2.8 ± 0.4 [2.0–3.8]	1.8 ± 0.2 [1.3–2.3]	1.55 ± 0.01						
Synechococcus cf. *nidulans* TAU-MAC 3010	3.5 ± 0.7 [2.1–5.3]	2.5 ± 0.3 [1.6–3.2]	1.4 ± 0.1						
Jaaginema sp. TAU-MAC 0110	6.8 ± 1.3 [4.2–10.6]	3.7 ± 0.4 [2.7–4.5]	1.83 ± 0.06						
Jaaginema sp. TAU-MAC 0210	8.8 ± 1.8 [5.6–13.6]	3.9 ± 0.6 [5.6–13.6]	2.25 ± 0.4						
Jaaginema sp. TAU-MAC 2210	9.2 ± 1.9 [5.0–13.5]	2.7 ± 0.6 [1.6–3.9]	3.4 ± 0.2						
Limnothrix redekei TAU-MAC 0310	8.0 ± 1.9 [4.8–15.5]	3.3 ± 0.3 [2.7–4.3]	2.4 ± 0.5						
Nodosilinea sp. TAU-MAC 0104	5.4 ± 1.1 [3.6–8.1]	2.7 ± 0.3 [2.1–4]	2 ± 0.6						
Anabaena cf. *oscillarioides* TAU-MAC 0199	4.5 ± 0.9 [2–5.8]	4.9 ± 1.0 [3.7–6.9]	0.19 ± 0.07	12.6 ± 0.8 [10.5–13.8]	11.4±1.0[9.3–13.2]	1.09 ± 0.03	19 ± 1.8 [18–25]	6.1 ± 0.4 [5–7]	3.11 ± 1.7
Chlorogloeopsis fritschii TAU-MAC 0599	10.0 ± 1.5 [7.2–13.8]	9.5 ± 1.4 [7.0–12.6]	1.05 ± 0.4	2.5 ± [2.1–3.7]	3.1 [2.7–4.2]	0.8 ± 0.3			
Desmonostoc muscorum TAU-MAC 0699	8.0 ± 1.0 [6–10.9]	6.8 ± 1.0 [4.7–9.6]	1.17 ± 0.08	10.1 ± 1.7 [6.4–14.4]	7.4 ± 0.9 [5.7–8.9]	1.36 ± 0.4	6.1 ± 0.05 [5.73–6.38]	5.1 ± 0.1 [4.60–5.82]	1.19 ± 0.04
Nostoc elgonense TAU-MAC 0299	8.7 ± 1.8 [5.4–12.5]	6.1 ± 0.8 [4.9–8.1]	1.3 ± 0.3	9.1 ± 1.9 [6.3–13.2]	6.8 ± 1.2 [5.4–10.2]	1.3 ± 0.2	5.9 ± 1.3 [5.00–6.47]	5.1 ± 0.5 [4.48–5.82]	1.15 ± 0.7
Nostoc oryzae TAU-MAC 2610	7.7 ± 0.7 [6.3–10.1]	7.1 ± 0.7 [5.0–9.4]	1.08 ± 0.2	8.8 ± 0.9 [7.3–11.0]	8.2 ± 1.0 [6.6–10.3]	1.07 ± 0.1	7.3 ± 1.2 [6.61–11.3]	3.8 ± 0.70.2 [3.00–4.48]	1.92 ± 1.1
Nostoc oryzae TAU-MAC 2710	8.6 ± 1.5 [5.4–11.3]	5.4 ± 0.5 [4.1–7.5]	1.59 ± 0.03	9.4 ± 1.5 [6.1–12.5]	7.0 ± 0.8 [5.5–8.7]	1.34 ± 0.7	5.6 ± 0.9 [4.8–6.7]	4.8 ± 0.1 [4.1–5.7]	1.16 ± 0.5
Nostoc sp. TAU-MAC 0799	9.4 ± 1.3 [6.4–12.5]	6.7 ± 0.4 [5.7–7.7]	1.40 ± 0.1	10.8 ± 1.4 [8.4–12.8]	9.1 ± 1.0 [7.4–10.8]	1.18 ± 0.5	7.1 ± 0.7 [6.4–7.9]	5.5 ± 0.4 [4.15–6.2]	1.29 ± 0.4
Nostoc sp. TAU-MAC 0899	9.2 ± 1.1 [7.2–11.7]	8.1 ± 0.8 [6.7–10.2]	1.13 ± 0.5	10.2 ± 0.8 [8.7–12.1]	9.5 ± 0.8 [8.1–11.2]	1.07 ± 0.3	6.1 ± 0.5 [5.56–6.85]	3.5 ± 0.01 [3.13–3.95]	1.74 ± 0.1
Trichormus variabilis TAU-MAC 2510	8.0 ± 1.2 [5.7–11.3]	6.9 ± 0.8 [4.6–8.6]	1.15 ± 0.5	7.6 ± 1.3 [5.6–10.3]	6.9 ± 1.1 [5.4–9.1]	1.10 ± 0.6	4.9 ± 1.1 [4.7–5.3]	4.3 ± 0.04 [3.6–5.1]	1.13 ± 0.6
Calothrix epiphytica TAU-MAC 0399	10.5 ± 2.0	7.7 ± 1.5	1.36 ± 0.8	11.8 ± 2.3	8.4 ± 1.4	1.40 ± 0.6			

*average ± standard deviation values, minimum–maximum values in brackets

Table S2. GenBank accession numbers for TAU-MAC strains used in the phylogenetic analysis.

Strain	Accession Number (16S rRNA)	Accession Number (ITS region)	Accession Number (*cpcBA*-IGS)
Microcystis aeruginosa TAU-MAC 0610	MN062656	MN062627	MN087417
Microcystis flos-aquae TAU-MAC 0410	MN062657	MN062628	MN087418
Microcystis flos-aquae TAU-MAC 1410	MN062658	MN062629	MN087419
Microcystis flos-aquae TAU-MAC 1510	MN062659	MN062630	MN087420
Microcystis flos-aquae TAU-MAC 1610	MN062660	MN062631	MN087421
Microcystis flos-aquae TAU-MAC 2010	MN062661	MN062632	MN087422
Microcystis viridis TAU-MAC 1810	MN062662	MN062633	MN087423
Microcystis sp. TAU-MAC 0710	MN062663	MN062634	MN087424
Microcystis sp. TAU-MAC 1710	MN062664	MN062635	MN087425
Microcystis sp. TAU-MAC 2110	MN062665	MN062636	MN087426
Microcystis sp. TAU-MAC 2310	MN062666	MN062637	MN087427
Microcystis sp. TAU-MAC 2410	MN062667	MN062638	MN087428
Synechococcus sp. TAU-MAC 0499	MN062668	MN062639	MN087429
Synechococcus cf. *nidulans* TAU-MAC 3010	MN062669	MN062640	MN087430
Jaaginema sp. TAU-MAC 0110	MN062670	MN062641	MN087431
Jaaginema sp. TAU-MAC 0210	MN062671	MN062642	MN087432
Jaaginema sp. TAU-MAC 2210	MN062672	MN062643	MN087433
Limnothrix redekei TAU-MAC 0310	MN062673	MN062644	MN087434
Nodosilinea sp. TAU-MAC 0104	MN062674	MN062645	MN087435
Anabaena cf. *oscillarioides* TAU-MAC 0199	MN062675	MN062646	MN087436
Chlorogloeopsis fritschii TAU-MAC 0599	MN062676	MN062647	MN087437
Desmonostoc muscorum TAU-MAC 0699	MN062677	MN062648	MN087438
Nostoc elgonense TAU-MAC 0299	MN062678	MN062649	MN087439
Nostoc oryzae TAU-MAC 2610	MN062679	MN062650	MN087440
Nostoc oryzae TAU-MAC 2710	MN062680	MN062651	MN087441
Nostoc sp. TAU-MAC 0799	MN062681	MN062652	MN087442
Nostoc sp. TAU-MAC 0899	MN062682	MN062653	MN087443
Trichormus variabilis TAU-MAC 2510	MN062683	MN062654	MN087444
Calothrix epiphytica TAU-MAC 0399	MN062684	MN062655	MN087445

Table S3. PCR primers used the phylogenetic analysis of cyanobacteria strains of TAU-MAC culture collection.

Primer	Target-gene	Sequence (5′–3′)	Size (bp)	Reference
PCbF	*cpcBA*-IGS	GGCTGCTTGTTTACGCGACA	720	[1]
PCaR_mod		CCAGTTCCACCAGCAATCAG		[2]
Cya106F	16S-23S rRNA	CGGACGGGTGAGTAACGCGTGA	1850	[3]
23S30R		CTTCGCCTCTGTGTGCCTAGGT		[4]

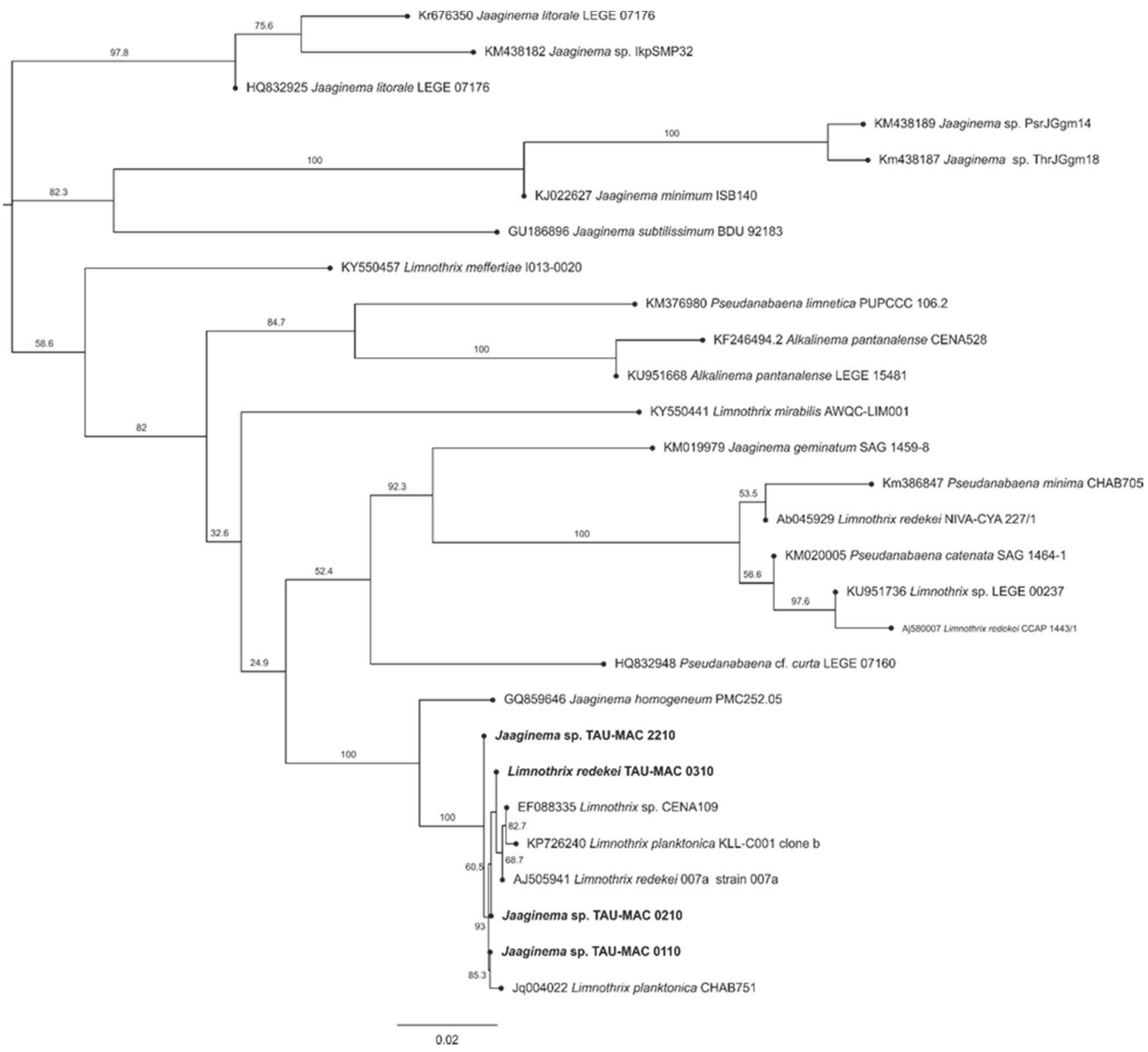

Figure S1. Phylogenetic consensus tree for *Jaaginema* and *Limnothrix* spp. based on 16S-23S rRNA and *cpcBA-IGS* sequences of TAU-MAC strains, reconstructed using the Maximum-Likelihood (ML) analysis. Numbers above branches indicate the bootstrap value (as percentages of 1,000 replications) for ML method. Strains of the present study are indicated in bold. Bar represents 0.020 nucleotide substitutions per site.

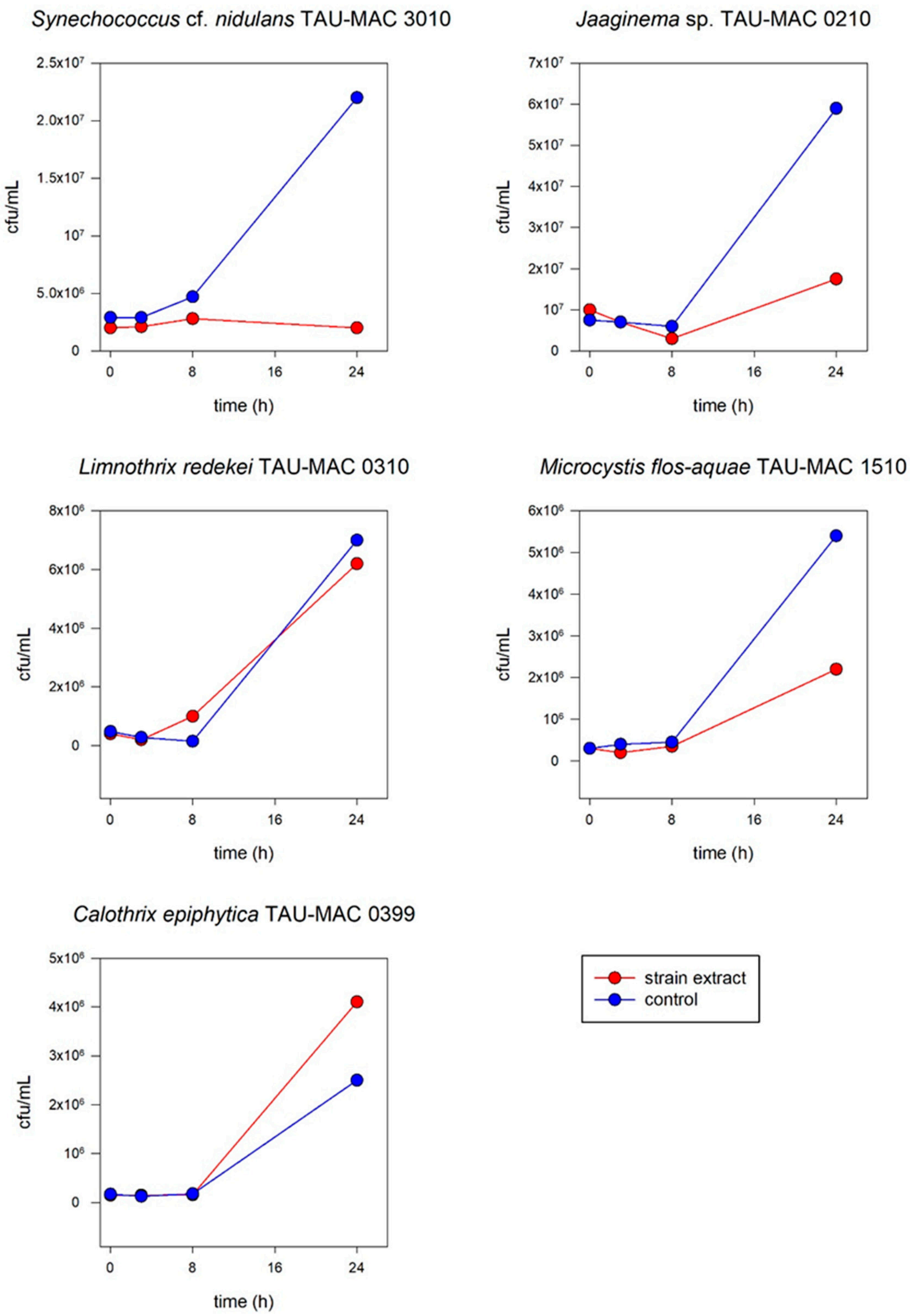

Figure S2. Inhibition of *Staphylococcus aureus* 9518 growth by the five cyanobacteria strains that showed clear inhibition zones.

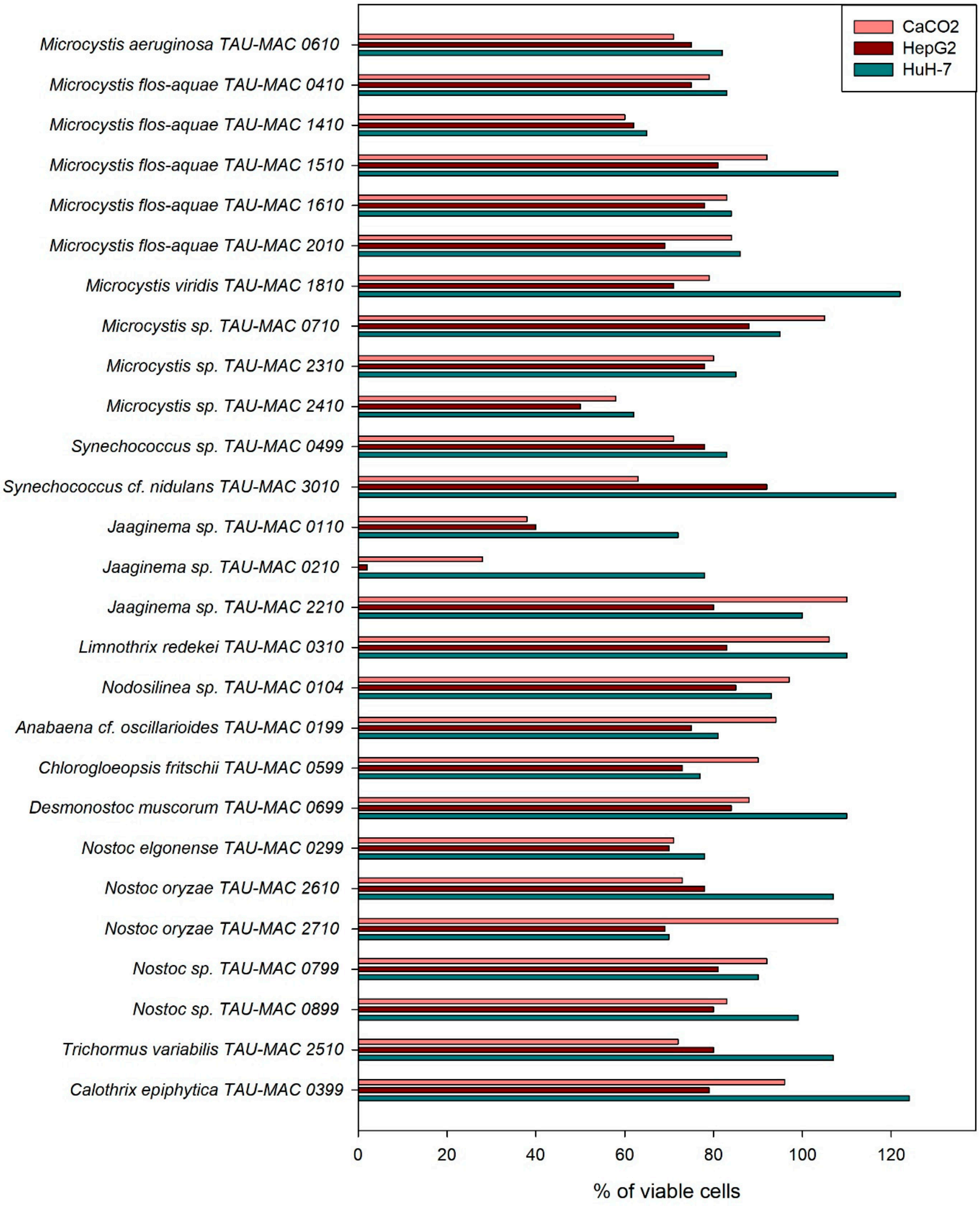

Figure S3. Cytotoxicity induced by aquatic extracts of the studied strains against Caco2, Huh-7, and HepG2 carcinoma cell lines after 24 h exposure.

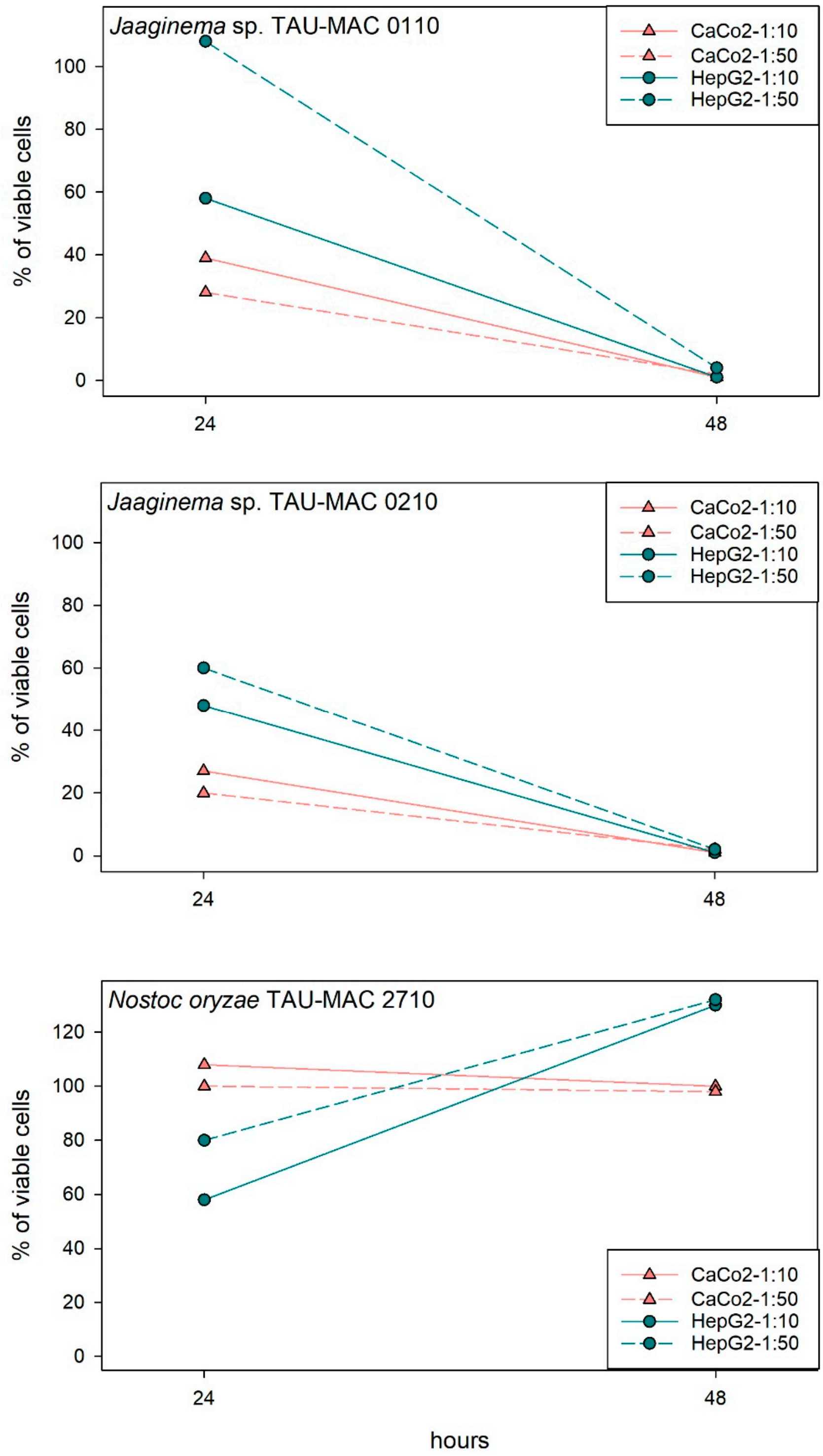

Figure S4. Cytotoxicity induced by methanolic extracts of three cyanobacterial strains against Caco2 and HepG2 carcinoma cell lines after 24h and 48h exposure in 1:10 and 1:50 dilutions.

References

1. Neilan, B.A.; Jacobs, D.; Goodman, A.E. Genetic diversity and phylogeny of toxic cyanobacteria determined by DNA polymorphisms within the phycocyanin locus. *Appl. Environ. Microbiol.* **1995**, *61*, 3875–83.
2. Manen, J.-F.; Falquet, J. The *cpcB-cpcA* locus as a tool for the genetic characterization of the genus *Arthrospira* (Cyanobacteria): evidence for horizontal transfer. *Int. J. Syst. Evol. Microbiol.* **2002**, *52*, 861–867.
3. Nübel, U.; Muyzer, G.; Garcia-pichel, F.; Muyzer, G. PCR primers to amplify 16S rRNA genes from cyanobacteria. *Microbiology* **1997**, *63*, 3327–3332.
4. Lepère, C.; Wilmotte, A.; Meyer, B. Molecular Diversity of *Microcystis* Strains (Cyanophyceae, Chroococcales) Based on 16S rDNA Sequences. *Syst. Geogr. Plants* **2000**, *70*, 275.
